# Self-medication practice among pregnant and postpartum women attending the regional hospital center of Souss Massa, Morocco: a cross-sectional study

**DOI:** 10.3389/fphar.2023.1233678

**Published:** 2024-01-08

**Authors:** Afaf Bouqoufi, Laila Lahlou, Fatima Ait El Hadj, Said Boujraf, Mohammed Abdessadek, Youssef Khabbal

**Affiliations:** ^1^ Laboratory of Innovation Research in Health Sciences, Team of Therapeutic Innovation, Translational Research and Epidemiology, Faculty of Medicine and Pharmacy, Ibn Zohr University, Agadir, Morocco; ^2^ Medical and Clinical Pharmacology Department, University Hospital Center Sous Massa, Agadir, Morocco; ^3^ Faculty of Medicine and Pharmacy, Sidi Mohamed Ben Abdellah University, Fez, Morocco; ^4^ Laayoune Higher School of Technology, Ibn Zohr University, Laayoune, Morocco

**Keywords:** gestational age, maternal and child health, OTC medication, pregnancy, pregnant women, self-medication, therapeutic use gestational age, therapeutic use

## Abstract

**Background:** Self-medication among pregnant women represents a serious risk to the mother’s and child’s health. It is a global concern that requires careful attention from professionals in healthcare. In Morocco, there is a lack of available data on self-medication and predicting variables among pregnant and postpartum women. The purpose of this study was to determine the incidence of self-medication and the factors that contributed to it among pregnant and postpartum women in the Sous Massa Regional Hospital.

**Methods:** A cross-sectional study was conducted using a pretested questionnaire among 420 pregnant and postpartum women who were attending the regional hospital center of the Sous Massa region from April to December 2022. Statistical analysis was performed using Jamovi Software. The logistic regression analysis was used to determine the significance of the association between the outcome and independent variables.

**Results:** The research enrolled 420 pregnant and postpartum women. During the current pregnancy, 24.8% of the women used self-medication. The leading common causes/symptoms that necessitate self-medication among pregnant and postpartum women were Anemia (84.8%), epigastralgia (16.8%), vomiting, pyrosis (15.2%), and urinary and vaginal infections The therapeutic families concerned with self-medication practice were Analgesics (41.4%), Antacids (20.3%), antimicrobials (13.5%), and Vitamin supplements (9%). According to the findings, the most frequent sources of information were pharmacists (45.6%), followed by physicians (44.3%). The primary reasons given by respondents for self-medication were the need for rapid release (51.7%), previous treatments with the same drugs (31.7%), and 20% reported difficulty of access to healthcare professionals. Out of 95.9% of the participants reported that they knew the dangers of self-medication and 96% of them were informed and received information about the dangers and contraindications of self-medication during pregnancy. This was significantly statistically associated with self-medication respectively with *p*-value = 0.031 and *p*-value = 0.005.

**Conclusion:** The findings of the present study provide an initial awareness of the state of self-medication among pregnant and postpartum women attending the regional hospital centers. It is recommended that healthcare professionals increase their interventions to improve the consciousness of pregnant women; this might require implementing suitable strategies to regulate the commercialization, delivery, and use of conventional medications.

## Introduction

Self-medication involves users treating their diseases with officially permitted drugs without obtaining previous medical advice and is defined as the use of commercial or homemade drugs without a doctor’s prescription in an attempt to treat symptoms or self-diagnosed medical conditions (World Health Organization, 2000). It is accepted as a kind of self-care by patients who depend on medical practitioners to diagnose or prevent illnesses using over-the-counter or prescription-only medications (Hughes et al., 2001). This therapy practice poses a significant risk of drug interactions, polypharmacy, misdiagnosis, inappropriate dose, extended usage, wrong drug selection, uncommon but severe side effects, dependency or addiction, and increasing antimicrobial resistance (Hardon and Geest, 1987; World Health Organization, 2000; Hughes et al., 2001). The number of individuals who self-medicate varies wildly by country. In a research study conducted in the United States, for instance, around 71% of men and 82% of women reported using self-medication at least once (Hong et al., 2005). In Africa, Tanzania is one of the African nations where self-medication is prevalent (Nsimba and Rimoy, 2005; Mangesho et al., 2007). The use of medications according to doctor’s prescriptions or instructions has been the subject of several studies. As well, various investigations have pointed to the incidence of self-medication during pregnancy, either in Africa or elsewhere (Araujo et al., 2013; Yimam et al., 2015; Cabut et al., 2017; Beza, 2018; Liao et al., 2018; Grover et al., 2019; Haque et al., 2020; Ake et al., 2021; Bobga et al., 2021) or worldwide (Lupattelli et al., 2014; Mohseni et al., 2018). Health policies, treatment accessibility, and medication distribution regulations are the major factors that make the difference between countries (Branthwaite and Pechère, 1996).

One of the susceptible demographic groups that use one of the two types of self-medication (80% Over-the-counter (OTC) and 20% prescription) is pregnant women (Stanley et al., 2019). It is because pregnancy is one of the most sensitive periods in a woman’s life that it is associated with physiological changes leading to many problems including nausea, vomiting, constipation, and gastrointestinal disorders in the first trimester. Pregnant women self-medicate by using drugs to relieve sympathetic signs of pregnancy (John and Shantakumari, 2015). In this context, it should be noted that pregnancy is a distinct physiological situation that requires specific consideration when taking drug treatment. However, the use of drugs without medical advice during pregnancy can have serious structural and functional consequences on the health of the mother and the development of the fetus, this could also cause an abortion and death of the fetus (Koren et al., 1998; Benjamin, 2003). According to the Food and Drug Authority (FDA), classification wishes to classify drugs in categories about safety in pregnancy from class A (the safest) to class D or X (the teratogenic group), Unfortunately, only a limited number of drugs (40%) are included in this group, showing that, because self-medication is a prevalent practice among pregnant women, Only a small number of drugs are expected to be harmless during pregnancy (Koren et al., 1998).

The high prevalence of self-medication in the last few years has caused physiological disorders and other health problems in pregnant women (Araujo et al., 2013). Moreover**,** to prevent all forms of illness brought on by self-medication, healthcare providers involved in prenatal supervision (primary care, gynecologists-obstetricians, and midwives) must contribute to the reduction of these dangers by monitoring and increasing awareness. It is crucial to assess self-medication among pregnant women, and close monitoring of drug use in a pregnant population is essential to preserve both the pregnant woman and the fetus. The limited studies reported self-medication and associated factors among pregnant women in Morocco, therefore in our work, we estimated the prevalence of self-medication and prescription drug use. We evaluated the associated factors of self-medication among pregnant women at Hassan II, the Regional Hospital Center in Sous Massa’s region, Morocco.

## Materials and methods

### Study area

This is a cross-sectional study carried out at the maternity unit of the Regional Hospital Center Hassan II of Sous Massa, which is the largest and only Regional Hospital in the Sous Massa region and has approximately 2.8 million inhabitants (HCP, 2020a). The Sous Massa region is experiencing a significant improvement in medical coverage and health facilities, but it has not yet fully met the needs of the population, given its demographic and geographical dimensions. Indeed, most of the hospital units and medical staff are located in the prefectures of Agadir Ida Outanane, and Inzegane Ait Melloul, as there is also a large disparity between urban and rural areas. In 2020, the Sous Massa region’s health infrastructure will comprise 1 specialized hospital and 8 general hospitals, with 17 delivery beds in urban areas and 58 in rural areas (HCP, 2020b). The hospital was selected because it represents the city’s whole population and its geographical situation. Another very important reason is that the regional hospital Hassan 2 is the only one in the Sous Massa region, and therefore it takes in all pregnant women who have risky pregnancies or deliveries.

### Study design

This is a cross-sectional, descriptive, and analytical study of women in the Obstetrics and Gynecology Unit at the regional hospital center Hassan II conducted in the region of Sous Massa. This study was conducted from April to December 2022 to assess self-medication practices and consumption of prescribed drugs among pregnant and postpartum women.

### Sample size and sampling method

We used the prevalence of self-medication in pregnant women from a previous study conducted in Morocco (Chergaoui et al., 2022). The sample size was calculated by using the assumption that for the proportional variable, the level of acceptable error is 5% (d = 0.05), and the expected proportion in the population is 32% (*p* = 0.32). At the 5% Type I error rate (i.e., *α* = 0.05), the sample size of the survey is 335. The initial sample size determined was three hundred thirty-five pregnant women. Based on the premise that 10% of participants would not respond, the sample size was increased to 369 to account for any missed data or non-response rate to ensure validity and reliability (Cochran, 1977; Bartlett et al., 2022).

In this survey, we have used random sampling. Every day, we go to the gynecology and obstetrics department to register new cases of patients. And then, we compile a database list of all pregnant and postpartum women who have visited the department in the last 24 h. This list is entered into Excel, and using the random function, the list of participants is extracted.

The total number of participants (369) in the study was also a factor in determining the daily sample size. For instance, the total sample size was 369 participants, and the study was conducted from April 2022 to December 2022 (except August, the annual holiday), an average of 10–15 participants were selected randomly each day (Except weekends and some religious and national holidays). The number of participants varied based on factors such as the period of the study; for example, from September to December, the number of participants that arrived each day was very low, with an average of 12–20 patients, and in April and July, the number was high, with an average of 30–35 patients.

### Population, inclusion, and exclusion criteria

The study used a comprehensive survey design, including all pregnant women who presented themselves to the Obstetrics and Gynecology Unit at the regional hospital center Hassan II either for prenatal consultation (PNC) or to give birth. Inclusion criteria: All voluntary and consenting pregnant or postpartum women presented for prenatal consultation at all frameworks of the system of primary care medical centers, and women admitted to the maternity unit at regional hospital center Hassan II. Exclusion criteria were women who declined to take part in this study or expressed a willingness not to participate in the study.

### Questionnaire

Data were collected using a structured questionnaire developed in French and translated into the Moroccan local dialect (Darija). We used a questionnaire that has already been developed in similar and previous studies (Courrier et al., 2013; HAL, 2023; Hamadi, 2023). The questionnaire was created and reviewed by a panel of clinical pharmacology and biostatistics experts. For content validity, a primary survey was used to conduct a pilot study. The pilot study aimed to measure the respondents’ comprehension of the questions. The survey was distributed in person by a single interviewer.

The reliability of our questionnaire measurement scale has been verified, to ensure validity and reliability using Cronbach’s alpha using Jamovi software. The value of Cronbach’s alpha was 0.763, and the reliability level was good and acceptable (Cortina, 1993).

The questionnaire was divided into five sections. The first section included questions about the participants’ socioeconomic and demographic characteristics, such as age, residency, level of education, income level, occupation, and social security. The second section is related to pregnancy and covers topics such as topics as sports, diet, smoking, and alcohol intake. It also includes the reason for the consultation and the time of pregnancy. The third part of the questionnaire covered information related to the consumption of prescribed drugs, the common illnesses associated with these uses, and the therapeutic families of drugs used. The final section reported the prevalence of self-medication among the participants, the common illness for this use, the therapeutic families of drugs used in self-medication, the reasons for use, and their sources of information.

### Operational definitions

#### Social protection in Morocco

The Moroccan social protection program provides for all employees in the public and private sectors. It protects against the risks of sickness, maternity, disability, old age, survival, death, and unemployment and provides family benefits. The National Medical Insurance Agency (ANAM) is responsible for the technical supervision of compulsory health insurance (AMO) and the Medical Assistance Scheme (RAMED), the management of which has been entrusted to the National Social Security Fund (CNSS), for persons subject to the social security system and their dependents, as well as for private sector pensioners. A basic medical coverage scheme, the Medical Assistance Scheme (RAMED), has been set up as part of national solidarity to cover the most deprived populations. To benefit from this scheme, the members of the household must have an annual income equal to or less than 3 767 MAD in urban areas and 5 650 MAD in rural areas (CESE, 2022).

#### Diet

Concerning food intake in pregnant women, we asked the participants about their diet during pregnancy. They could describe their diet as healthy (homemade food) or mixed (fast food).

Hence, using the following definition, we considered the Mediterranean Diet to be a healthy meal:

Excessive consumption of extra virgin (cold pressed) olive oil, vegetables, such as leafy green veggies, fruits, cereals, nuts, and pulses/legumes; modest consumption of fish and other meat; dairy products; and red wine; and low consumption of eggs and sweets are among the definitions. Every description gives an idea of how often, daily, or biweekly these items should be taken, as well as the amounts that should be included in the diet. Most lack explicit recommendations for serving sizes or quantities, and they do not state how much of any food additives, including sauces, condiments, tea, coffee, salt, sugar, or honey, should be included in the diet. According to certain definitions, cereals must contain primarily whole grains (Davis et al., 2015).

#### Sports activities

We considered the participants physically active if they had done the physical activity or walked for at least 30 min three times a week (Espass, 2022).

#### Malformation

Since the discovery of real-time ultrasonography in the clinical setting in the 1970s, prenatal ultrasound diagnoses of congenital malformations have been documented. Fetal abnormalities may now be identified more accurately and earlier in the gestational period because of improvements in the clarity of these ultrasound pictures. The features of the abnormality determine when a prenatal diagnosis should be made. The sooner and simpler a diagnosis may be established, the more divergent the morphological anomalies are. The biological development of the organs involved must also be taken into account in the diagnosis of congenital anomalies (Jstage, 2022).

### Judgment criteria

#### Definition of self-medication

Self-medication (SM) is the term for the practice of treating a disease without a prescription, under the supervision of a physician, or using Over-the-counter (OTC) to ensure the effective drug selection, decision, dosage, and treatment protocol (World Health Organization, 2000).

### Data collection procedure

Women who were eligible and accepted to take part in the study were asked to complete the questionnaire on their own. However, some women who could not read or write were interviewed by the interviewer to complete their surveys, using oral questions and written responses. The interviewer only read the questions for those women and avoided leading questions to avoid bias. When necessary, reasons were given to the women to ensure that they understood the questions. The study administrators contacted the subjects and explained the study’s goals. Then, an informed consent document was distributed to all participants, and those who signed it were provided with a printed copy of the questionnaires and were given time to complete them. Women who were pregnant in their first trimester provided information on self-medication during this period, those who were pregnant in their second and third trimesters released details on the first and second trimesters, and those who were pregnant in their third and final trimesters or were in term of childbirth provided information on the whole period of their pregnancy. The completed questionnaires were collected and stored in a secure location. The data was uploaded and saved into an Excel spreadsheet that was appropriately designed. Furthermore, before statistical analyses, the data were processed using best practices for raw data management to identify any inaccuracies or incompleteness.

### Ethical approval

The study protocol was approved by the ethics committee for biomedical research at the Faculty of Medicine and Pharmacy of Rabat, Morocco. Before commencement, the study was approved by the biomedical research ethics committee at Mohamed V University (68-21). All procedures were performed according to the revised Declaration of Helsinki. Each participant was also required to sign an informed consent form. These were well-written and stated the objective of the research. Confidentiality was assured by assigning each participant a code number for analysis. The women who participated in the research were identified by an anonymous study number assigned to each participant. There was no financial aid or awards offered, and there was no conflict of interest. Each respondent was given the option to decline participation in the study.

### Statistical and data analysis

Two authors Laila Lahlou and Afaf Bouqoufi conducted the statistical analysis. Statistical descriptive analyses were performed for the study volunteers. Continuous variables were summarized using the mean and standard deviation (SD), and proportions were used for categorical variables. Self-medication was the dependent variable. The independent variables were the socio-demographic characteristics of pregnant women and their practices. Comparisons were made using Fisher’s exact test and a Chi-square test. Statistical significance was considered at *p* < 0.05. The association between self-medication and socio-demographic characteristics was investigated using bivariate logistic regression analysis. A multivariate binary logistic regression model was used to identify the factors independently associated with modern drug self-medication practice during pregnancy for variables with a *p*-value less than 0.25. Odds ratios (OR) with 95% confidence intervals (CIs) were used to assess the strength of associations between each exposure variable and the dependent variable, and statistical significance was set at *p* = 0.05. Jamovi Software was used for all statistical analysis. To compare the practice of self-medication among pregnant and postpartum women before and after pregnancy, we used the Mac-Nemar test.

## Results

### Socio-demographic characteristics of the study respondents

In this study, 420 pregnant and postpartum women participated with a response rate of 100%. The mean age of the respondents was 28.7 (±6.35) years. Only 3 (0.7%) of the respondents were not Moroccan. Most 402 (95.7%) of the respondents were married and more than half 216 (51.4%) were from rural areas. Nearly two-thirds 278 (68.3%) of the participants were educated 51 (12.1%) were housewives and the rest were employed. The majority of the participants 303 (72.1%) have a low-income level and half of them 222 (52.9%) do not have medical insurance (Table 1).

**TABLE 1:** Socio-demographic characteristics of pregnant and postpartum women who practice Self-medication.

Variable	Overall N (%)	Self-medicated: No N (%)	Selfmedicated:YES N (%)	*p*-value
Age				
Mean ± SD	28.7 ± 6.35	28.6 ± 6.41	28.7 ± 6.22	0.918
Family situation				
Not Married	18 (4.3)	13 (4.1)	5 (4.8)	0.782
Married	402 (95.7)	303 (95.9)	99 (95.2)	
Origin				
Rural	216 (51.4)	168 (53.2)	48 (46.2)	0.215
Urban	204 (48.6)	148 (46.8)	56 (53.8)	
Nationality				
Not Moroccan	3 (0.7)	3 (0.9)	0 (0)	1.000
Moroccan	417 (99.3)	313 (99.1)	104 (100)	
Education				
Illiterate	133 (31.7)	101 (32)	32 (30.8)	0.821
Educated	278 (68.3)	215 (68)	72 (69.2)	
Employment				
Unemployed	51 (12.1)	37 (11.7)	14 (13.5)	0.635
Employed	369 (87.9)	279 (88.3)	90 (86.5)	
Income level				
Low	303 (72.1)	234 (74.1)	69 (66.3)	0.128
Middle	117 (27.9)	82 (25.9)	35 (33.7)	
Medical insurance				
None	222 (52.9)	164 (51.9)	58 (55.8)	0.493
Insured	198 (47.1)	152 (48.1)	46 (44.2)	
Parity				
Nulliparity	44 (10.5)	36 (11.4)	8 (7.7)	0.285
Parity	376 (89.5)	280 (88.6)	96 (92.3)	
Nutrition				
Healthy	312 (74.3)	239 (75.6)	73 (70.2)	0.271
Mixed	108 (25.7)	77 (24.4)	31 (29.8)	
Sport				
No	229 (54.4)	168 (53.2)	61 (58.7)	0.329
Yes	191 (45.5)	148 (46.8)	43 (41.3)	
Pregnancy Age				
First trimester	17 (4)	16 (5.1)	1 (1)	0.280
Second trimester	12 (2.9)	10 (3.2)	2 (1.9)	
3rd trimester	96 (22.9)	72,822.8)	24 (23.1)	
Postpartum	295 (70.2)	218 (69)	77 (74)	
Malformation				
No	416 (99)	315 (99.7)	101 (97.1)	**0.049**
Yes	4 (1)	1 (0.3)	3 (2.9)	
Premature				
None	416 (99)	313 (99.1)	103 (99)	1.000
Yes	4 (1)	3 (0.9)	1 (1)	
Miscarriage				
None	405 (96.4)	302 (95.6)	103 (99)	0.130
Yes	15 (3.6)	14 (4.4)	1 (1)	
Gestational diabetes				
None	346 (82.4)	264 (83.5)	82 (78.8)	0.275
Yes	74 (17.6)	52 (16.5)	22 (21.2)	
Hypertension				
None	383 (91.2)	289 (91.5)	94 (90.4)	0.738
Yes	37 (8.8)	27 (8.5)	10 (9.6)	
Pregnancy at Risk				
No	129 (30.7)	94 (29.7)	35 (33.7)	0.454
Yes	291 (69.3)	222 (70.3)	69 (66.3)	
Pregnancy Follow up				
Not followed	110 (26.2)	77 (24.4)	33 (31.7)	0.138
Well followed	310 (73.8)	239 (75.6)	71 (68.3)	
Prescribed drugs				
No	30 (7.2)	28 (8.9)	2 (1.9)	**0.017**
Yes	388 (92.8)	286 (91.1)	102 (98.1)	
Do you think you know the dangers of self-medication?				
No	16 (4.1)	8 (2.7)	8 (8.5)	**0.031**
Yes	372 (95.9)	286 (97.3)	86 (91.5)	
Have you received information about the dangers of self-medication during pregnancy?				
No	16 (4)	7 (2.3)	9 (9.4)	**0.005**
Yes	380 (96)	293 (97.7)	87 (90.6)	

The bold values are statistically significant.

### Obstetric related characteristics

A total of 295 (70.2%) of the respondents were postpartum and 125 (29.8%) of the respondents were pregnant. Only (10.5%) of the respondents are null parity and most of them (89.5%) had more than one child. Nearly two-thirds (69.3%) of the women reported having experienced health issues during their pregnancies and that their pregnancy was risky, 17.6% had gestational diabetes, 1% respectively for malformation and premature, and 3.6% reported that they had a miscarriage. Overall, the largest 310 (73.8%), monitored their pregnancy (Table 1).

### Prevalence of self-medication among pregnant and postpartum women

The prevalence of self-medication among pregnant and postpartum women pregnancy was 24.8% with a CI of 95% (20.9%–29.1%). The majority of the participants (74%) practiced self-medication in the postpartum stage and 26% were in the pregnancy period (Table 1). In addition to self-medication practice, 388 (92.8%) of participants reported the use of prescribed drugs during their pregnancy, which was statistically significantly associated with self-medication with *p*-value = 0.01 as shown in Table 1.

### Self-medication practice according to the trimester

**FIGURE 1:**
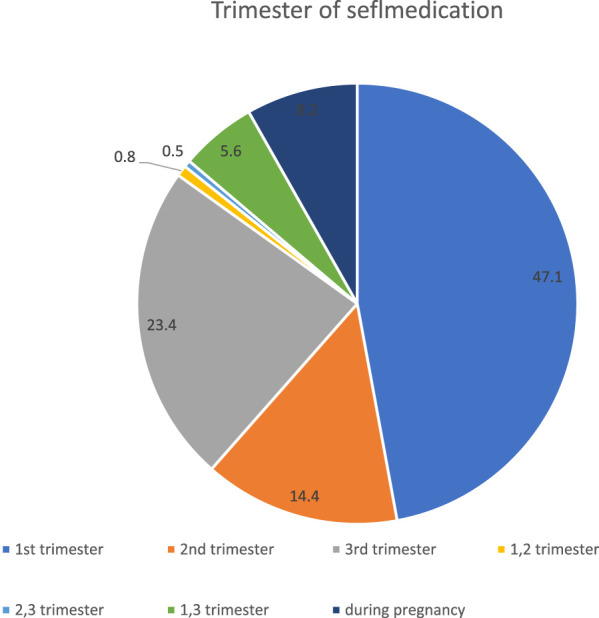
Selfmedication practice according to the trimester of pregnant women.

In this survey, 47.1% of the participants self-medicated in the first trimester, and 23.4% of the participants reported practicing self-medication in the third trimester. 8.2% of pregnant women use self-medication at any time in their pregnancies (Figure 1).

### Predictors of self-medication practice

When bivariate logistic regression was performed, malformation, prescribed drugs, Knowledge about the risks of self-medication, and data collected regarding the risks of self-medication were the determining factors for self-medication. The practice of self-medication among pregnant and postpartum women increases the risk of malformation (*p* = 0.054; OR = 9.356; 95% CI, 0.963–90.949).

Pregnant and postpartum women who have used prescribed drugs were more likely to practice self-medication (*p* = 0.03*; OR = 4.993; 95% CI, 1.168–21.334). Pregnant and postpartum women who have no knowledge and did not receive the information about the danger of self-medication were more likely to practice self-medication (*p* = 0.02; OR = 0.301; 95% CI, 0.01–0.825/(*p* = 0.005; OR = 0.231; 95% CI, 0.0836–0.638) respectively (Table 2).

**TABLE 2:** Logistic regression analysis to identify predictors for self-medication among pregnant and postpartum women attending Hospital Center Hassan 2.

Characteristic	Crude odds ratio	CI 95%	*p*-value	OR adjusted	CI 95%	*p*-value
Family situation	0.850	0.295–2.44	0.762	—	—	—
Married-not married						
Origin	1.324	0.849–2.065	0.215	1.414	0.8657–2.31	0.166
Urban-rural						
Education	1.057	0.655–1.706	0.821	—	—	—
Educated-illiterate						
Employment	1.173	0.607–2.268	0.635	—	—	—
Unemployed- Employed						
Income Level	1.448	0.897–2.335	0.130	1.236	0.7215–2.12	0.441
Middle-Low						
Medical Insurance	0.856	0.548–1.336	0.856	—	—	—
Insured-None						
Parity	1.543	0.693–3.435	0.288	—	—	—
Parity- Nulliparity						
Pregnancy Age	1.282	0.778–2.112	0.329	—	—	—
Postpartum-Pregnant						
Malformation	9.356	0.963–90.949	**0.054**	27.661	1.4445–529.67	**0.028**
Yes-no						
Premature	1.013	0.104–9.845	0.991	—	—	—
Yes-no						
Miscarriage	0.209	0.027–1.612	0.133	0.230	00,230–2.30	0.210
Yes-no						
Gestational diabetes	1.362	0.781–2.377	0.277	—	—	—
Yes-no						
Hypertension	1.139	0.531–2.440	0.738	—	—	—
Yes-no						
Pregnancy at Risk	0.835	0.520–1.339	0.454	—	—	—
Yes-no						
Pregnancy Follow up Not following-Following	0.693	0.426–1.127	0.140	1.588	0.9286–2.71	0.091
Prescribed drugs	4.993	1.168–21.334	**0.030**	4.851	0.9951–23.65	0.051
Yes-no						
Do you think you know the dangers of self-medication?	0.301	0.01–0.825	**0.020**	0.666	0.1527–2.90	0.588
Yes-no						
Have you received information about the dangers of self-medication during pregnancy?	0.231	0.0836–0.638	**0.005**	0.251	0.251	0.065
Yes-no						

The bold values are statistically significant.

#### Adjusting on the following variables

Origin, Income level, Malformation, Miscarriage, Pregnancy follow-up, and prescribed medication. The multivariable logistic regression model revealed that only malformation was significantly associated (*p* < 0.05) with self-medication practice during pregnancy. Self-medication by pregnant and postpartum women increases the risk of malformation (AOR = 27.661, 95% CI: 1.4445–529.67) (Table 2).

### Common causes and drugs commonly among pregnant and postpartum women

The leading common causes/symptoms that necessitate self-medication among pregnant and postpartum women attending the regional hospital center of Sous Massa were Anemia 328 (84.8%), epigastralgia 65 (16.8%), vomiting and pyrosis 59 (15.2%). Urinary, vaginal infections 58 (15%), vaginal Itch (pruritus) 42 (10.9%), and 3 (2.3%) Anxiolytic (Table 3).

**TABLE 3:** Common causes and Diseases/symptoms related to pregnancy that have forced pregnant and postpartum women to self-medicate or use prescribed drugs.

Common diseases and symptoms related to pregnancy	n (%)^a^
Epi, Gastralgia	65 (16.8)
Vomiting, pyrosis	59 (15.2)
Leucorrhoea, metrorrhagia	14 (3.6)
Pelvic and articular pain	39 (10.1)
Flu, Cold, Cough	11 (2.8)
Anemia	328 (84.8)
Urinary, Vaginal infections	58 (15)
Headache fevers	24 (6.2)
Vaginal Itch (pruritus)	42 (10.9)
Intestinal gas, Constipation	19 (4.9)
Other^b^	64 (16.5)

^a^ Total percentages exceed 100% due to the selection of more than one source.

^b^ ^:^ Other diseases that are mentioned by the participants (Asthma, Diabetes, Hypertension, Anxiety, Cardiovascular disease, Pregnancy stabilization).

Regarding the therapeutic families concerned with self-medication practice, 55 (41.4%) of the participants self-administered analgesics, 27 (20.3%) Antacids, 18 (13.5%) antimicrobials and 12 (9%) Vitamins. On the other side, several respondents disclosed using some medications as prescribed drugs, the majority of the participants 301 (87%) used Iron, 83 (24%) used vitamins, 45 (139%) used antibiotics, and 10 (2.9%) used cough and cold remedies (Table 4).

**TABLE 4:** Therapeutic families of common Drugs used in self-medication and for prescription among pregnant and postpartum women.

Therapeutic families of common Drugs used	Prescribed drugs users n (%)^a^	Self-medication users n (%)^a^
Analgesics	38 (119)	55 (41.4)
Antibiotics	45 (139)	13 (9.8)
Antipyretics	9 (2.6)	14 (10.5)
Cough and Cold Remedies	10 (2.9)	13 (9.8)
Antacids	50 (14.5)	27 (20.3)
Antiemetic	41 (11.8)	12 (9)
Antimicrosis	23 (6.6)	18 (13.5)
Anxiolytic	0	3 (2.3)
Iron	301 (87)	0
Vitamins	83 (24)	12 (9)
Other^b^	49 (14.2)	15 (11.3)

^a^ Total percentages exceed 100% due to the selection of more than one source.

^b^ ^:^ Other class therapies mentioned by the participants (AINS, AIN, antispasmodic, laxatives, antiasthmatic, Progesterone, Polycystic ovarian disease, Antihypertensives).

### Source of information and self-medication reasons of study participants

Among pregnant and postpartum women who practice self-medication during their current pregnancy, the most common sources of information were pharmacists 45.6%, 44.3% were physicians, and 8.9% were friends and family. According to the place of procuration, 83.1% of the participants got the drugs from the pharmacy (drug store), 10.8% from their neighbors, and 7.7% from their families. The main reasons mentioned by the participants for self-medication were the need for fast release (51.7%), followed by previous experiences with the same drugs (31.7%), and finally, 20% of the participants reported that they have difficulties accessing healthcare professionals (Table 5). Globally, this study showed that 372 (95.9%) pregnant and postpartum women reported that they knew the dangers of self-medication. Also, 380 (96%) of the participants were informed and received information about the dangers and contraindications of self-medication during pregnancy. This was significantly statistically associated with self-medication, respectively, with *p*-values of 0.031 and 0.005, as shown in Table 1.

**TABLE 5:** Self-medication reasons, Source of information, and place of procuration of study participants.

	Number (N)	Percentage (%)
Place of procuration		
Drug Store (Pharmacy)	54	83.1
Family	5	7.7
Surrounding	7	10.8
Grocery store	1	1.5
Source of information		
Pharmacist	36	45.6
Family/Friends	7	8.9
Physicians	35	44.3
Notice	2	2.5
Midwife	3	3.8
Reason of self-medication		
Drugs are accessible	7	11.7
Economic reasons	11	18.3
Need for fast release	31	51.7
Previous experiences	19	31.7
Difficulties in accessing Health professional	12	20

### Self-medication before and during pregnancy

A total of 238 pregnant and postpartum women (62.6%) reported practicing self-medication before and during pregnancy. Moreover, 37.4% of the participants in the study practiced self-medication during pregnancy. 181 of the participants (60.9%) practiced self-medication before pregnancy. The results of the McNemar test indicated that self-medication before and during pregnancy was significantly associated (*p* < 0.001) (Table 6).

**TABLE 6:** Distribution of absolute and relative frequency of self-medication before and during pregnancy.

Self-medication		During pregnancy		Total
		No: N (%)	Yes: N (%)	(N)
Before Pregnancy	No: N (%)	116 (39.1)	34 (37.4)	150
	Yes: N(%)	181 (60.9)	57 (62.6)	238
Total (N)		297	91	388

## Discussion

The present study aimed to assess the prevalence and factors associated with self-medication practice among pregnant and postpartum women attending the regional hospital center of Souss Massa. To our knowledge, this is the first study carried out in the south of Morocco. The prevalence of self-medication during pregnancy is 24.8% with a CI of 95% (20.9%–29.1%) which is high. These findings are lower than the findings of a study conducted in Settat, a Moroccan city located in the Casablanca-Settat region which is 32% (Chergaoui et al., 2022). The prevalence of self-medication found in our study is the same compared to other studies, 25.1% in Ethiopia (Abeje et al., 2015a), 27.7% in Brazil (Lutz et al., 2020), 21.9% in Mexico (Alonso-Castro et al., 2018), and 25.8% in Iran (Rafiee et al., 2018). Conversely, in developed countries, a multinational study reported a high prevalence of self-medication practice, 85% in the Netherlands, 82% in the United Kingdom, and 84% in Finland (Lupattelli et al., 2014). Another study conducted in French showed a very high prevalence of 72% (Cabut et al., 2017). Moreover, the most important factor relating to self-medication among pregnant women is education level. Illiterate pregnant women or incomplete primary or secondary education were more likely to practice self-medication than pregnant women with a high level of education (Abeje et al., 2015a; Jstage, 2022), these findings are broadly similar to numerous studies (Biritwum et al., 2000; Awad et al., 2005; Afolabi, 2008; Marwa K. et al., 2018) but are very different from the findings reported in developed countries in which a high level of education is associated with a high prevalence of self-medication.

This result may be explained by the fact that pregnant women in developed nations with a high level of education may quickly recognize the effects of medications by using information obtained from numerous sources, such as the Internet, but they also believe they can treat minor illnesses without contacting a doctor (Pereira et al., 2021). In contrast, illiterate pregnant women will not be able to understand the drug leaflets and the online information. Self-medication practice was also associated with the term of pregnancy, it was high during the first trimester, and as gestational age increased, the prevalence decreased (Marwa KJ. et al., 2018). The higher self-medication practice is due to the many symptoms that are related to the first trimester, including nausea, vomiting, fever, and headache. These findings reveal an extremely dangerous concern because drug exposure during this period is almost certain to result in congenital abnormalities as teratogenicity occurs during organogenesis in the first 3 months of gestational age (Nunes de Melo et al., 2006). However, this was caused by inadequate knowledge of the risks of self-medication during pregnancy (Alani et al., 2020), and regarding the healthcare system in some countries, pregnant women preferred self-medicate rather than consult a doctor, due to their difficulty accessing and time savings (Awad et al., 2005). Pregnant women thought it would take a long time to get the drugs from the healthcare institution. Additionally, they believed that their illness was related to pregnancy symptoms and frequently received treatment without obtaining medical advice. The decision of pregnant women to seek medical care and increase medication adherence will be heavily influenced by their perception of risk and beliefs about medication use. Although pregnant women’s knowledge and educated attitudes about the adverse effects of drugs are very important, mistaken or insufficient perception may lead to the unwarranted termination of pregnancy (Tefera et al., 2020). Moreover, in Iran, the main reason is that pregnant women had easy access to medication without a medical prescription and were prescribed excessive drugs (Baghianimoghadam et al., 2013). In this study, prior experience with the drug was one of the reasons for self-medication. The result is comparable to a study conducted in Ethiopia (Jambo et al., 2018).

This study also found that pregnant women who were self-medicated before pregnancy were more likely to self-medicate during pregnancy, with a significant associated *p*-value of 0.001. This indicates that some women do not care about the sensitivity of their pregnancy. In the current study, the most common illnesses/symptoms that necessitate self-medication among pregnant and postpartum women were anemia, morning sickness, gastrointestinal disorders, headache, cold and cough, and infection. It is because most of these illnesses are symptoms related to pregnancy, and most pregnant women suffer from colds, coughs, fevers, and infections due to physiological changes that cause a decrease in the immune defense, making pregnant women more susceptible to microbial, fungal, and viral infections.

Drugs commonly used in self-medication among pregnant and postpartum women were analgesics, antacids, antibiotics, and vitamin supplements. The use of drugs matches common illnesses compelling self-medication among pregnant women as discussed above. This type of drug is commonly used by pregnant women’s communities, these findings have already been observed in other studies (Nsimba and Rimoy, 2005; Araujo et al., 2013; Afshary et al., 2015; Gbagbo and Nkrumah, 2021; Chergaoui et al., 2022). The use of analgesics during pregnancy can be related to different disorders. According to the FDA, using paracetamol during pregnancy can be associated with neuropsychiatric risks, neurodevelopmental problems, autism spectrum disorders, and even attention-deficit/hyperactivity disorder (Brandlistuen et al., 2013). As a result, pregnant women should carefully consider using paracetamol and should always check with their doctors before taking it. A large proportion of pregnant women use antibiotics during pregnancy, antibiotic use during pregnancy has been discovered to affect the vaginal microbiome in pregnant women before delivery, with long-term implications on the newborn’s early microbial colonization and relation to childhood obesity (Kuperman and Koren, 2016). The use of antibiotics is a result of healthcare services not placing sufficient emphasis on informing new mothers about the possible side effects of antibiotics on the fetus and mother. It is concerning that pregnant women are using antibiotics without a prescription, and this situation has to be looked at more extensively to develop effective health promotion messages and measures. On the other side, several respondents disclosed using some medications as prescribed drugs, the majority of the participants (87%) used Iron. Iron and anti-anemia drugs were the most common pregnancy-related medications consumed by pregnant women in our study. The Moroccan healthcare system prescribes and delivers anti-anemia drugs for free to all pregnant women who come for antenatal consultations either in rural or urban health centers. This is because preventing blood loss is considered one of the main causes of maternal mortality (Leke et al., 2018).

In this current survey, for the majority, the main source of information regarding medication use among pregnant and postpartum women was pharmacists. This was also reported in Ethiopia, the United Kingdom, and Norway (Sawalha, 2007; Miní et al., 2012; Tefera et al., 2020). This again differs from some studies where husbands, family members, and neighbors were reported to be the main sources of information (Jambo et al., 2018). It could be a reason that in Morocco, pharmacists are often well regarded by the community, with many considering pharmacists as physicians. This makes it understandable why they were the main source of information for these pregnant women. The pharmacist should take this chance to inform the women about drug use and safety since they are frequently in direct contact with them when they purchase prescriptions. In other studies, pregnant women had another reason for considering the pharmacist as a source of information, it is simply because they were far away from healthcare facilities, and were more inclined to go straight to pharmacies and buy drugs without a prescription (Abeje et al., 2015b).

The participants also relied on the internet and media for knowledge regarding drugs. In this case, since all pregnant women today have access to the internet and social networks, these sources should be exploited to spread the message about the dangers of self-medication during pregnancy. Our study found that pregnant and post-partum women who had used drugs before pregnancy had a higher percentage of self-medication during pregnancy than those who had not used medications before pregnancy. Our findings are similar to those of a study conducted in Iran (Ebrahimi et al., 2017) demonstrated that people who had used drugs before pregnancy had a relatively higher odds ratio of self-medication during pregnancy than those who had not used drugs before pregnancy. The continuation of self-medication during pregnancy can be explained by the lack of consultation and access to healthcare providers; the researchers concluded that pregnant women continue to take drugs in the absence of prenatal medical supervision (Niriayo et al., 2021; Mohammed et al., 2023).

The present study’s findings provide knowledge about the state of self-medication among pregnant and postpartum women attending the regional hospital center of Souss Massa. It is recommended that healthcare professionals increase their awareness of pregnant women and implement suitable strategies to regulate the commercialization, delivery, and use of conventional medications. Self-medication should be avoided by providing information (public drug education) about the potential risks and dangers to the fetus and mother’s health to healthcare professionals (physicians, nurses, etc.) involved in pregnancy supervision.

## Study limitations

The limitation of the current study was that participants were not asked whether they had informed medical staff and physicians of their drug use. When making prescription decisions, doctors may use this information to take medication interactions and overdoses into consideration. Additionally, consider that the placement for the first sample was selected randomly and that the validity of reporting previous self-medication is sensitive to recall bias. Beginning pregnant women may not have had a chance to take drugs yet, which might have an impact on the reported outcomes for regularly prescribed prenatal medications. The study may also be affected by social desirability bias, which might result in an underestimation of drug usage among women and a confusing knowledge of drug names.

## Conclusion

Self-medication is a known major risk factor that might alter the mother’s and the fetal health threatening both lives. The findings demonstrated that self-medication is a common practice among pregnant women in the studied region of Morocco. While ANC follow-up was shown to be protective against self-medication practice. Hence, better maternal education and experiencing health issues throughout pregnancy were positively linked with self-medication. It is required to improve maternal health services to significantly reduce the risks. Without taking into account gestational age or types of faced health issues, all pregnant women attending ANC services must receive health education from governmental health institutions about the risks associated with self-administered medications. Pharmacists and drug retailers should contribute to not distributing drugs, including OTC medications, to pregnant women without carefully assessing the risks and doctors’ prescriptions. This study supports interested care contributors to fully investigate the self-use of industrialized drugs and herbal medicine by pregnant women. The effectiveness and safety of commonly used drugs and herbs by pregnant women should be considered to protect the health of the mother and fetus.

## Data Availability

The raw data supporting the conclusion of this article will be made available by the authors, without undue reservation.

## Abbreviations

AINS, Anti-inflammatory non steroids (Anti-inflammatoires non stéroïdiens); AIS, Anti-inflammatory steroids (Anti-inflammatoires stéroïdiens); AMO, Assurance Maladie Obligatoire (Obligatory health insurance); ANAM, National Health Insurance Agency (Agence Nationale de l'Assurance Maladie); ANC, Antenatal care; CI, Confidence Interval; CNSS, National Social Security Fund (Caisse Nationale de Sécurité Sociale); FDA, Food and Drug Authority; OPK, Polycystic ovary syndrome (PCOS); OTC, Over The Counter; RAMED, Medical Assistance Scheme (Régime d'Assistance Médicale); WHO, World Health Organization.
