# Supplementary material for: Self-medication practice among pregnant and postpartum women attending the regional hospital center of Souss Massa, Morocco: a cross-sectional study
Source: Front Pharmacol. 2024 Jan 8;14:1233678. doi: 10.3389/fphar.2023.1233678 (PMC10800875; doi:10.3389/fphar.2023.1233678)
Supplement: Supplementary file 1 [file Table1.DOCX]

Numéro d’ordre :

Date d’entrée :

**Identité**

1. **Age**: شحال في عمرك
2. **Situation Familiale** : 2 الحالة العائلية

• Célibataire عازبة

• Mariée متزوجة

•Divorcée مطلقة

•Veuve ارملة

1. **Origine des Femmes** 4 فين كتسكني

• Milieu urbain وسط حضاري

• Milieu Rural وسط قروي

1. **Nationalité :**

•Marocaine • Non marocaine

1. **Niveau de la scolarité** 3 واش قارية

• Primaire الابتدائي

• Collège الاعدادي

• Niveau Bac ou le baccalauréat نيفو باك او الباكالوريا

• Études supérieures الجامعة.

1. **Profession** 8 المهنة

• Professions intellectuelles supérieures (cadres) اطار عالي

•Professions intermédiaires Professions de santé موظف في الصحة

•Employées موظف

•Artisanes صانع تقليدي

•Ouvrières عاملة

• Sans activité بدون عمل

1. **Niveau socio-économique** 5 المستوى الاجتماعي الحالة المادية

• Bas • Moyen • Haut

1. **Sécurité social** 6. التغطية الصحية

• RAMED راميد

• Pas de couverture sociale بدون تغطية

• CNSS الضمان الاجتماعي

1. **La parité**:
2. 1 2 3 4 plus

**Habitudes et Grossesse**

9 **L’alimentation**. واش ماكلة ديالك متوازنة 10. **Activité physique** واش كتمارسي الرياضة

• Saine صحية • OUI نعم

• Mixte مختلطة • NON لا

11. **Alcool**  واش كتشربي 12. **Tabac** واش كتكمي

• OUI نعم • OUI نعم

• NON لا • NON لا

**Grossesse et prise médicamenteuse**

13. **Age de la grossesse** 13 شحال عندك في الحمالة ديالك

•1 er trimestre الفصل الأول

•2e trimestre الفصل التاني

•3e trimestre الفصل الثالت

14. **Pendant vos grossesses, avez-vous rencontré l’un des problèmes suivants ?**

اثناء الحمل واش لقيتي شي مشاكل

Fausse couche : اجهاض Malformation : تشوه خلقي Prématurité*:الخداج Diabète gestationnel : سكري الحمل

•OUI •OUI •OUI •OUI

•NON •NON •NON •NON

(* accouchement avant le début du 9è mois)

14. **Consultation et suivi de grossesse** واش متبعة على الحمل ديالك

• Bien suivie متابعة جيدة.

• Non suivie معمرك تبعتي عند طبيب

• Mal suivie متابعة غير جيدة

15. **Avez-vous eu recours à l'utilisation de médicaments durant votre grossesse?** واش فايت ليك خديتي شي دوا في فترة الحمل ديالك

• Oui نعم

• Non لا

16. **Pour quelle raison avez-vous utilisé ces médicaments ?** اشنوهو سبب لي خلاك تاخدي دوا

•Consultation Prénatale (CPN) استشارة قبل الولادة

• Céphalée + Douleur articulaire الم الرأس والم المفاصيل

•Fièvre السخانة.

•Douleur pelvienne الم الحوض او الكرش الصغيرة.

• Vertige الدوخة

•Leucorrhées الافرازات المهبلية.

•EPI gastralgie الم المعدة.

•Métrorragies نزيف الدم في المهبل

•Vomissement + Pyrosi التقيا وحرقة المع

•Toux الكحة

•Œdèmes des membres inferieurs انتفخاح واحمرار الأطراف.

•Anorexie. فقدان الشهية

• Autre ……………………………………

17. **A quel moment de votre grossesse le recours à ces médicaments a-t-il eu lieu?** اشمن فترة في حمالة ديالك خديتي دوا.

• Au cours du premier trimestre الفصل الأول

• Au cours du second trimestre الفصل التاني

• Au cours du dernier trimestreالفصل الثالث

18. **Avez-vous pris des médicaments de votre propre initiative et sans prescription médicale pendant votre grossesse?** واش هاد دوا خدتيه لراسك وبلا ميخرجوا ليك طبيب

• Oui نعم

• Non لا

19. **Si « Oui », veuillez citer le ou les médicaments que vous avez consommez en automédication:** …………………….……………………. ………………………………………… اشنو هما هاد الادوية لي خديتي

20. **Type des médicaments pris par les parturientes lors de la grossess**e نوع د الادوية لي خديتي

• Antalgiques (contre la douleur) دوا ديال الحريق أولا الوجع.

• Antibiotiques

•Antipyrétiques (contre la fièvre) دوا د سخانة.

•Antitussifs (contre la toux) دوا د كحة

•Laxatifs (constipation) دوا د قبط

•Anxiolytiques (contre l’anxiété) دوا د أعصاب

•Hypnotiques (troubles du sommeil) دوا د نعاس

•Antiacides (contre brulure d’estomac et remontées acides) دوا د حرقة ديال المعدة.

• Anti-rhume. دوا د رواح

•Antiémétiques (nausées, vomissements) دوا ديال الردان والتقيا.

• Antispasmodiques (maux de ventre) دوا د وجع في الكرش

• AINS مضادات الالتهاب قويلبات ظهر مثلا

21. **Lieu de procuration** البلاصة لي خديتي منها الدوا

• Entourage محيط • Epicerie العطار • Famille العائلة • Pharmacie الصيدلة

22. **Pour quelle raison n'êtes-vous pas allée consulter votre médecin traitant, obstétricien ou sage-femme avant de prendre l'initiative d'utiliser le médicament**? علاش ممشيتيش تشوفي طبيب ديالك قبل متاخدي دوا

• Accès aux médicaments دوا كاين عندك

• Raisons économiques أسباب اقتصادية

• Besoin de soulagement rapide. بحاجة الى راحة سريعة.

• Difficultés d'accès à un professionnel de santé. صعوبة الوصول الى الطبيب او مختص الصحة

• Expérience précédente. من خلال التجربة السابقة لي كانت عندك

23. **Vers quel personnel de santé ou autre vous tourne-vous pour obtenir des informations sur les médicaments que vous utilisez ?** شكون لي مشيتي عندو باش تاخدي معلومات على دوا

• Médecin traitant الطبيب لي متبعة معاه

• Gynécologue /Obstétricien اخصائي التوليد وامراض النساء.

• Sagefemme القابلة

• Pharmacien الصيدلي

• Notice de médicament. نشرة الدواء

• Internet الانترنيت

• Famille /Amis العائلة او الأصدقاء

• Autre …………………

24. **Pensez-vous connaitre les dangers de l’automédication?** واش عندك علم بمخاطر التطبيب الذاتي

• Oui نعم • Non لا

•Vous pensez ne pas avoir de connaissances suffisantes واش كظني انو ماعندكش معلومات كافية

25. **Avez-vous reçu des informations concernant les dangers en cas d’automédication ou les médicaments à éviter au cours du suivi de votre grossesse?** واش كانو عندك معلومات بخصوص مخاطر التطبيب الذاتي او تعرفي الدوا لي ممنوع على المراة الحاملة

• Oui نعم • Non لا.

26. **Hors grossesse, avez-vous recours à l’automédication?** قبل ماتكوني حاملة واش كاتخدي ادوية لراسك وبدون استشارة د طبيب

• Oui نعم • Non لا

27. **Pensez-vous avoir eu une modification de votre comportement relatif à l'automédication pendant votre grossesse?** واش كتحسي انه تبدلات فيك شي حاجة من بعد ماخديتي دوا في فترة الحمل

• Oui نعم • Non. لا

**28.Quel type de traitement préférez-vous prendre pendant la grossesse ?**

•Médicaments • Médecine tradionnelle • le deux

Je vous remercie d’avoir consacré un peu de temps pour répondre à ce questionnaire.
