# Supplementary material for: Self-medication practice among pregnant and postpartum women attending the regional hospital center of Souss Massa, Morocco: a cross-sectional study
Source: Front Pharmacol. 2024 Jan 8;14:1233678. doi: 10.3389/fphar.2023.1233678 (PMC10800875; doi:10.3389/fphar.2023.1233678)
Supplement: Supplementary file 2 [file Table2.DOCX]

Number of Order:

Date of Entry:

Identity

1. **Age**: How old are you?
2. **Marital Status:**

• Single

• Married

• Divorced

• Widowed

1. **Women's Origin: Where do you live?**

• Urban Area

• Rural Area

1. **Nationality:**

• Moroccan

• Non-Moroccan

1. **Education Level: What is your educational level?**

• Primary School

• Middle School

• High School (Baccalauréat)

• Higher Education (University)

1. **Profession: What is your occupation?**

• Higher Intellectual Professions (Executives)

• Intermediate Professions, Health-related Professions

• Employees

• Artisans

• Workers

• Unemployed

1. **Socio-economic Level: What is your socio-economic status?**

• Low

• Middle

• High

1. **Social Security: Which type of health coverage do you have?**

• RAMED

• No Social Coverage

• CNSS

1. **Parity:**

0 1 2 3 4 plus

**Habits and Pregnancy**

1. **Nutrition: Is your diet balanced**? YES NO
2. **Physical Activity: Do you engage in physical activity?**

• Yes

• No

1. **Alcohol: Do you consume alcohol?** YES NO
2. **Tobacco: Do you smoke?** YES NO

**Pregnancy and Medication Use**

1. **Pregnancy Stage: How far along are you in your pregnancy?**

• 1st trimester

• 2nd trimester

• 3rd trimester

1. **Pregnancy Problems: Have you experienced any of the following issues during your pregnancies?**

• Miscarriage

• Malformation

• Prematurity

• Gestational Diabetes

1. **Pregnancy Consultation: Are you regularly consulting a healthcare professional during your pregnancy?**

• Well-followed

• Not followed

• Poorly followed

1. **Medication Use During Pregnancy: Have you used any medications during your pregnancy?**

• Yes

• No

1. **Reason for Medication Use: What was the reason for using these medications?**

Prenatal Consultation (CPN)

• Headache + Joint Pain

• Fever

• Pelvic Pain

• Dizziness

• Leucorrhoea

• Gastralgia

• Metrorrhagia

• Vomiting + Heartburn

• Cough

• Lower Limb Edema

• Anorexia

• Other..

1. **Timing of Medication Use: At which stage of your pregnancy did you use these medications?**

• 1st trimester

• 2nd trimester

• 3rd trimester

1. **Self-Medication: Have you taken any medications without a medical prescription during your pregnancy?**

• Yes

• No

1. **Medication Types: What types of medications did you take during pregnancy?**

Painkillers (against pain

• Antibiotics

• Antipyretics (against fever))

• Antitussives (against cough) –

• Laxatives (for constipation)

• Anxiolytics (against anxiety) –

• Hypnotics (for sleep disorders) –

• Antacids (against heartburn and acid reflux) –

• Cold medicine

• Antiemetics (nausea, vomiting)

• Antispasmodics (stomach cramps)

• NSAIDs (Non-Steroidal Anti-Inflammatory Drugs)

1. **Source of Medication Procurement: Where did you get the medication from?**

• Around

• Grocery Store

• Family

• Pharmacy

1. **Reasons for Not Consulting a Doctor Before Self-Medicating: Why didn't you consult a doctor before using medication?**

• Access to medications

• Economic reasons

• Need for quick relief –

• Difficulties accessing a healthcare professional –

• Previous experience -

1. **Source of Information on Medications: From whom do you seek information about the medications you use?**

• Primary care physician

• Gynecologist / Obstetrician

Midwife

Pharmacist

Medication leaflet

• Internet

• Family / Friends

• Other

1. **Awareness of Dangers of Self-Medication: Do you know the dangers of self-medication?**

• Yes

• No

• You think you lack sufficient knowledge.

1. **Information on Medication Safety During Pregnancy: Have you received information about the dangers of self-medication and medications to avoid during pregnancy?**

• Yes

• No

1. **Self-Medication Outside of Pregnancy: Do you practice self-medication when not pregnant?**

• Yes

• No

1. **Behavior Change Regarding Self-Medication During Pregnancy: Do you think there has been a change in your self-medication behavior during pregnancy?**

• Yes

• No

1. **Preferred Treatment During Pregnancy:**

• Medications

• Traditional Medicine

• Both

**Thank you for taking the time to answer this questionnaire**.
